# Anti-adipogenic effects of *Tropaeolum majus* (nasturtium) ethanol extract on 3T3-L1 cells

**DOI:** 10.1080/16546628.2017.1339555

**Published:** 2017-06-14

**Authors:** Gi-Chang Kim, Jin-Sook Kim, Gyoung-Mi Kim, Song-Yi Choi

**Affiliations:** ^a^ Department of Agrofood Resources, National Academy of Agricultural Science, Rural Development Administration, Wanju-gun, Republic of Korea

**Keywords:** Edible flower, obesity, PPARG, SREBF1, CEBPA

## Abstract

**Background**: Edible flowers, Tropaeolum majus has been used as a disinfectant and an antibiotic, and for wound healing, but the anti-obesity effects of this plant have not been reported previously

**Objective**: We investigated the anti-adipogenic effects of *T. majus* ethanol extract (TME) on 3T3-L1 cells.

**Design**: 3T3-L1 cells were differentiated in the presence of different concentrations of TME. Lipid accumulation levels were determined using Oil-Red O staining and a triglyceride content assay. Changes in the expression of proteins related to adipocyte differentiation in 3T3-L1 cells were determined by SDS-PAGE and western blotting.

**Results**: The highest inhibition of lipid accumulation was observed at a TME concentration of 300 µg/mL. Additionally, TME concentrations ranging from 20 µg/mL to 500 µg/mL led to a decrease in the expression of adipocyte differentiation regulators, peroxisome proliferator-activated receptor γ, CCAAT element binding protein α, and sterol regulatory element binding transcription factor 1. This decrease was shown to be concentration-dependent.

**Discussion**: Taken together, the results of this study demonstrate that TME inhibits lipid accumulation and reduces the expression PPARG, CEBPA, and SREBF1, which regulate adipocyte differentiation in 3T3-L1 cells.

**Conclusions**: TME may be a potential novel therapeutic agent for the prevention and treatment of obesity.

## Introduction

The number of obese individuals is growing worldwide, and the National Cholesterol Education Program Adult Treatment Panel III [1] and the World Health Organization (WHO) have declared obesity as one of the key features of metabolic syndrome [2]. Obesity is defined as an increase in body weight caused by the excessive accumulation of adipocytes, and it usually accompanies other conditions, such as insulin resistance-related diabetes, hypertension, and dyslipoproteinemia [3]. These diseases are exacerbated by the adverse effects of obesity on blood pressure regulation, insulin sensitivity, and plasma triglyceride and leptin concentrations [4]. Furthermore, multiple recent studies have suggested obesity to be closely associated with the inflammatory responses linked to insulin resistance in type 2 diabetes [5], as well as with the diversity of the gut microbiota [6].

Lipid accumulation, a major cause of obesity, occurs as a result of increased triglyceride production and increased adipocyte differentiation in a process termed adipogenesis [7]. The differentiation of preadipocytes to mature adipocytes is regulated by the expression of genes involved in adipogenesis [8], and the most investigated adipogenic genes are peroxisome proliferator-activated receptor-γ (PPAR*G*), CCAAT/enhancer-binding proteins (CEBPs), and sterol regulatory element-binding transcription factor 1 (SREBF1), all of which are transcription factors and key regulators of adipogenesis [9]. PPARG binds to the peroxisome proliferator-activated receptors (PPARs), a type of nuclear receptor, and it plays a role in the regulation of adipogenesis while promoting lipogenesis in mature adipocytes [10,11]. The C/EBP family comprises proteins characterized by a C-terminal leucine zipper domain and a basic DNA-binding domain, and inhibition of their expression results in a reduction of lipogenesis in adipocytes and a decrease in the body weight in obesity-induced mice [12]. SREBP1, one of the three isoforms of SREBF, promotes the biosynthesis of fatty acids [13]. Additionally, it was demonstrated that the accumulation of triglycerides is inhibited in SREBF1c^–/ –^ mice fed a high-fat diet (HFD) [14].

Edible flowers have been used as supplements to improve the presentation and smell of prepared food. Recently, they have attracted attention as rich sources of phytochemicals, which are beneficial for human health. Flowers, found in a multitude of colors, contain a variety of phenolic compounds, such as flavonoids and anthocyanin, which are natural antioxidants [15]. Several studies have investigated the anti-cancer activities [16], anti-inflammatory properties [17], and anti-mutagenic activities [18] of edible flowers containing high levels of antioxidants, and other bioactive properties of these flowers are currently being analyzed. *Tropaeolum majus*, a member of the Tropaeolaceae family, is a native plant of the Andes, found from Bolivia to Colombia. Since the Ministry of Food and Drug Safety (MFDS) in Korea approved the use of flowers, leaves, and shoots of *T. majus* as food ingredients [17], it has been commercially grown in several farms. *T. majus* has outstanding antioxidant activity due to its rich phenolic content, which includes anthocyanin and ascorbic acid [19]. Furthermore, it contains high levels of glucotropaeolin, an aromatic glucosinolate and a precursor of aromatic isothiocyanate shown to have anti-cancer properties [20–22]. Additional properties of *T. majus*, including antibiotic and expectorant activities [23,24], antiscorbutic activities [25], and antimicrobial and anti-inflammatory activities [26], have been investigated as well.

In light of previous findings, which demonstrated various effects on health and the abundance of phenolic compounds in *T. majus*, we investigated the anti-obesity effects of *T. majus* extracts. To this end, mouse 3T3-L1 cells were differentiated in the presence of different concentrations of *T. majus* ethanol extracts (TMEs). Adipocyte differentiation was determined on the basis of lipid accumulation, as measured by Oil-Red O staining and triglyceride quantification assay, and the expression of proteins associated with adipogenesis (PPARG, CEBPs, and SREBF1) was examined using western blotting.

## Materials and methods

### Chemicals and reagents

*T. majus* was purchased from the Angel Farm (Gongju, Korea) in the spring of 2015. 3T3-L1 cells were purchased from the American Type Culture Collection (ATCC, Manassas, VA, USA). Dulbecco’s Modified Eagle Medium (DMEM), dexamethasone (DEX), 1-methyl-3-isobutyl xanthine (IBMX), insulin from bovine pancreas (INS), formaldehyde, and isopropanol were purchased from Sigma-Aldrich (St. Louis, MO, USA), whereas fetal bovine serum (FBS) and bovine serum were purchased from Gibco (Grand Island, NY, USA). MTS Cell Viability reagents were purchased from Promega (WI, USA). Rabbit polyclonal anti-PPARG, anti-CEBPA, and mouse polyclonal anti-SREBF1 antibodies were provided by Abcam (UK). West-Q Chemiluminescence (ECL) detection kit and horseradish peroxidase-conjugated anti-rabbit IgG and anti-mouse IgG secondary antibodies were purchased from GenDepot (USA). Polyvinylidene fluoride (PVDF) membranes were obtained from Bio-Rad (Hercules, CA, USA).

### TME extraction

**Figure 1.**
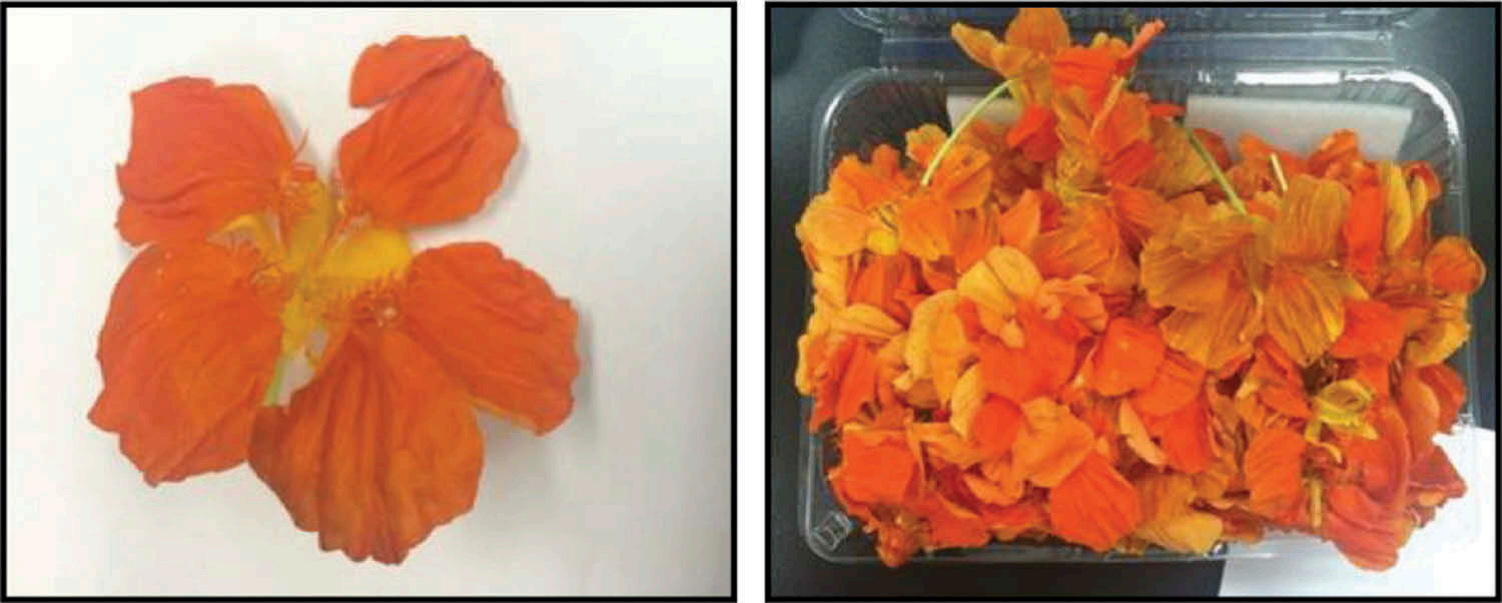
Images of Tropaeolum majus (nasturtium).

After removal of the pistil, stamen, and sepal, *T. majus* flowers (Figure 1) were washed twice with distilled water and dried at 25°C. Subsequently, they were ground in a homogenizer (IKA, Germany) with 95% ethanol and the extraction was performed in a darkroom for 24 h. The ethanol extract was concentrated in a rotary vacuum evaporator at 40°C and freeze-dried (Ilshin, Korea) for 5 days. The freeze-dried powder was stored at −20°C.

### Cell culture and TME treatment

Preadipocytes were cultured in DMEM containing 10% FBS and 1% penicillin/streptomycin (P/S; Sigma, USA) in an incubator at 37°C with 5% CO_2_/95% air. To induce differentiation, the cells were seeded at 1.25 × 10^5^ cells/well in a 6-well plate. The medium was replaced on day 2, and the cells reached full confluence by day 4, when they were treated with 10% FBS and MDI solution (0.5 mM 3-isobutyl-1-methylxanthine, 1 µM dexamethasone, and 5 µg/mL insulin) to induce differentiation. Furthermore, to observe effects of TME treatment on adipocyte differentiation, TME was dissolved in dimethyl sulfoxide (DMSO) and added to each well in different concentrations (10, 25, 50, 75, or 100 µg/mL). At day 2 after the initiation of differentiation, the culture medium was replaced with DMEM containing TME sample, 10% FBS, 1% P/S, and 5 µg/mL insulin. At 4 days after the initiation of differentiation, the culture medium was replaced with fresh DMEM containing the TME samples, 10% FBS, and 1% P/S every 2 days. Fully differentiated adipocytes at day 8 after the induction of differentiation were used for further experiments.

### MTS assay

Following the treatment of cells, cytotoxicity was determined using the 5-(3-carboxy methoxyphenyl)-2H-tetrazolium inner salt (MTS) assay [14], which measures the activity of mitochondrial dehydrogenases that convert MTS to formazan. Culture medium was removed from the wells on the final day of the induction of differentiation, and MTS Cell Viability reagents were added to the medium containing 10% FBS. After 4 h of incubation, the absorbance at 490 nm was measured, and cell viability in each well was expressed as a percentage of the absorbance measured in wells containing cells treated with ethanol (control group). Wells containing only medium, without cells, were used for the determination of concentration-specific absorbance values (blanks).

### Oil-Red O staining

After removal of the culture medium, the cells were washed twice with phosphate-buffered saline (PBS). They were then fixed with 10% formaldehyde at 25°C and rinsed 3 times with PBS. To stain the lipids in the adipocytes, the cells were treated with filtered Oil Red O solution for 1 h at 25°C and rinsed twice with PBS. The resulting red-stained lipid droplets were observed microscopically and extracted with isopropanol. The absorbance was measured at 540 nm to quantify the residual fat content within the adipocytes.

### Triglyceride quantification assay

The triglyceride content was measured in adipocytes treated with TME and the control cells. Culture medium, collected after the removal of cells, was analyzed using a LabAssay Triglyceride kit (AM1575-K, Asan Pharmaceuticals, Korea) to quantify the residual triglycerides.

### Immunoblotting

The 3T3-L1 cells induced to differentiate were suspended in radioimmunoprecipitation assay buffer (RIPA; R4100, GenDepot, USA) at 4°C for 30 min, and the resulting solution was centrifuged at 14,000 rpm for 30 min to obtain cell lysates.

Cell lysates were analyzed using a protein assay kit (BR500-022, Bio-Rad, USA) to measure protein contents, and these samples were further analyzed using 10% SDS-PAGE (BR456-8033, Bio-Rad, USA). After separation on SDS-PAGE, the proteins were transferred to a PVDF membrane (BR170-4156, Bio-Rad, USA), which was blocked with 5% skim milk for 1 h. Primary anti-PPARG (1:1000), anti-CEBPA (1:500), anti-SREBF1(1:1000), and anti-GAPDH (1:2500) antibodies were added, and the samples were incubated for 15 h at 4°C. Subsequently, the membranes were hybridized with the secondary horseradish peroxidase-conjugated anti-rabbit IgG (SA002-500) or anti-mouse IgG (SA001-500, GenDepot) antibodies for 1 h at 25°C. Finally, the membranes were incubated with the chemiluminescent substrates, and the protein bands were imaged using the ChemiDoc MP imaging system (Bio-Rad, USA).

### Statistics

All experiments were performed in triplicate, and the results are presented as the mean value ± standard deviation. The data were statistically analyzed using Student’s t-test in SPSS statistical software, with p < 0.05 considered statistically significant.

## Results

### Effects of TME on adipocyte viability

**Figure 2.**
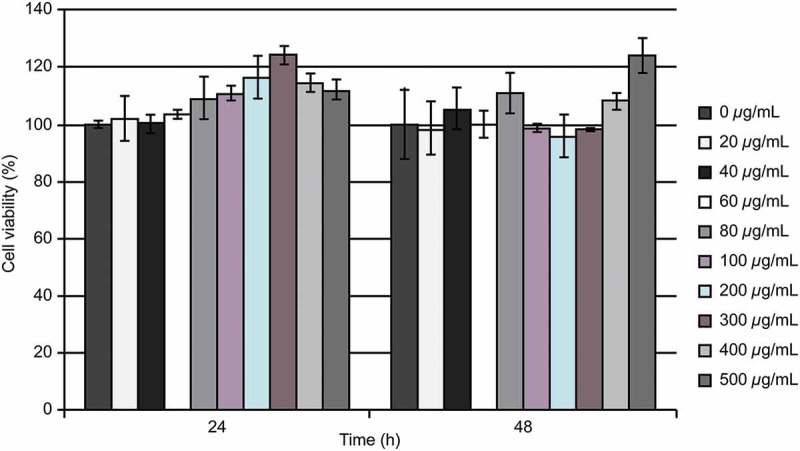
TME effects on 3T3-L1 cells after 24 or 48 h of treatment. The obtained results were averaged and expressed as the percentage of the control.

Cell viability was analyzed following the incubation of 3T3L1 cells with TME at concentrations of 20–500 µg/mL for 24 or 48 h. At all concentrations, cell viability was shown to be above 80% of the control, untreated sample (Figure 2). Based on these results, the concentrations of 20, 300, and 500 µg/mL TME were selected for further experiments.

### TME treatment inhibits lipid accumulation

**Figure 3.**
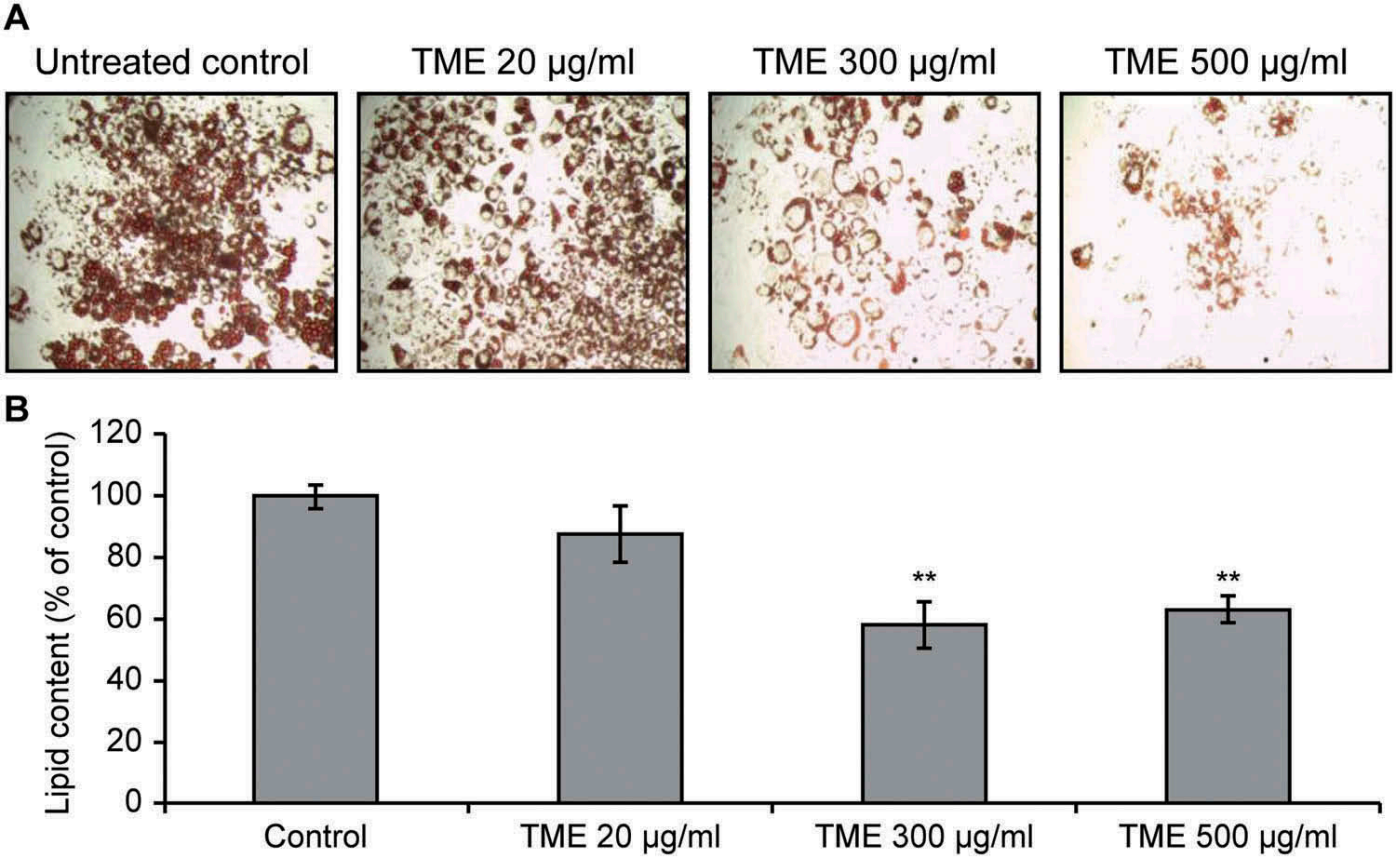
Effects of TME treatment on the accumulation of lipids in 3T3-L1 cells. (A) Oil-Red O staining of the treated cells and the controls at day 8 following the induction of differentiation and TME treatment. (B) Quantification of lipid content in the treated cells. **P < 0.01, compared with the control (Student’s t-test).

**Figure 4.**
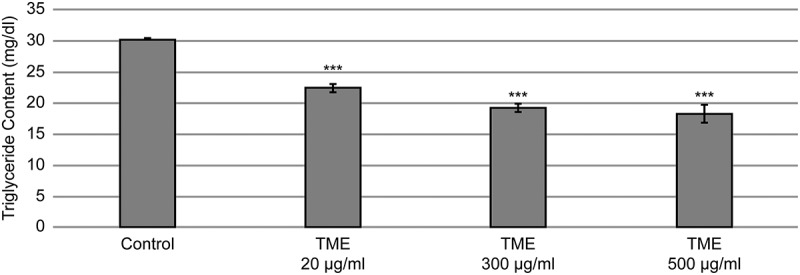
Effect of TME treatment on triglyceride concentration in the differentiated 3T3-L1 cells. Cells were differentiated in the absence or presence of TME for 8 days, and triglyceride content was determined. ***P < 0.001, compared with the control (Student’s t-test).

Morphological changes were detected in TME-treated 3T3-L1 cells, which changed from a pre-differentiation spindle-like shape to a round shape after the differentiation. Furthermore, microscopic observations of the Oil Red O-stained lipid droplets within the cells revealed a concentration-dependent decline in the lipid content in the TME-treated cells, in comparison with that observed in the control group (Figure 3). Absorbance measurements showed that the accumulation of lipids in 3T3-L1 adipocytes decreased in a concentration-dependent manner, with the lowest lipid accumulation rate observed in the cells treated with 300 µg/mL TME. In Figure 4, the results for triglyceride quantification in the differentiated 3T3-L1 adipocytes treated with different concentrations of TME are presented, showing that the TME treatment leads to a reduction in triglyceride content. This reduction ranged from 25.8% to 54.7%, and the concentration of 500 µg/mL TME was shown to have the greatest effects on the reduction of triglyceride content.

### Effects of TME treatment on the expression of adipocyte-specific transcription factors during adipocyte differentiation

**Figure 5.**
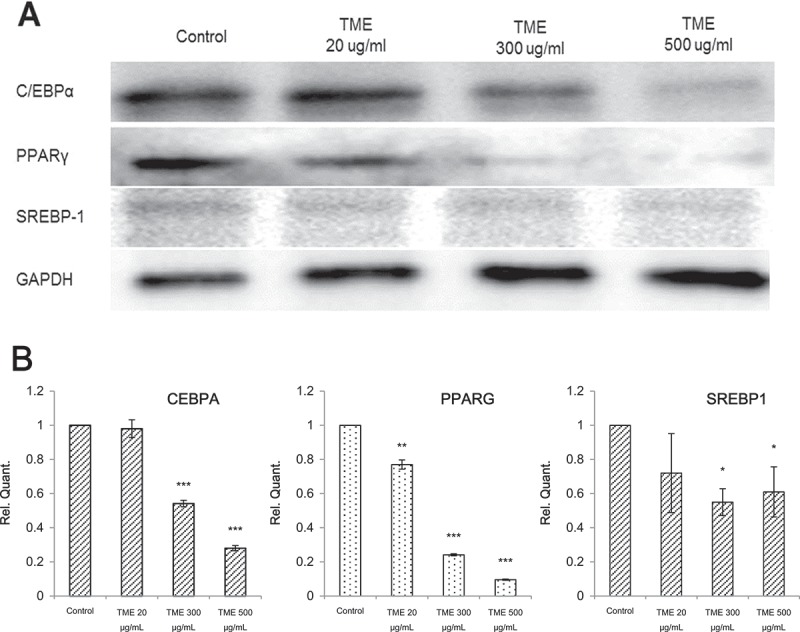
Effect of TME on the expression of the regulators of adipocyte differentiation. 3T3-L1 cell differentiation was induced the presence or absence of different concentrations of TME. (A) Representative immunoblots, showing the changes in the expression of CEBPA, PPARG, and SREBF1. (B) Densitometric analysis of CEBPA, PPARG, and SREBF1expression. *P < 0.05; **P < 0.01; ***P < 0.001.

The expression levels of PPARG, CEBPA, and SREBF1 were analyzed using western blotting, and it was demonstrated that TME treatment leads to a decrease in PPARG, CEBPA, and SREBF1 expression in a concentration-dependent manner, in comparison with that in the control samples (Figure 5). TME treatment significantly decreased PPARG expression levels by 23.0%–90.4%, whereas CEBPA expression was significantly decreased following the treatment with 300 µg/mL and 500 µg/mL TME (45.8% and 71.9%, respectively) in comparison with the control. Treatment with 20 µg/mL TME led to only a slight decrease in CEBPA expression. In SREBF1 level, there was no significant difference between treatment with 20 µg/mL TME and control. However, treatment with 300 and 500 µg/mL TME led to a significant decrease in the expression levels of SREBF1 protein, in comparison with that in the control.

## Discussion

The aim of this study was to investigate the anti-adipogenic activities of *T. majus*, a plant used as a food item in Korea, in 3T3-L1 adipocytes. TME treatment was shown to decrease lipid accumulation and to inhibit the expression of PPARG, CEBPA, and SREBF1, which are transcription factors involved in the regulation of adipogenesis pathway, in 3T3-L1 adipocytes. Expressed in the early stages of adipocyte differentiation, PPARG is a nuclear receptor that plays an important role in adipogenesis, and adipogenic thiazolidinedione is a high-affinity ligand for this receptor [27]. PPARG also regulates adipocyte phenotypes [28], and it induces the activation of various signaling pathways by binding to X-receptors (RXR), forming PPAR-RXR heterodimers, and regulating the transcription of target genes that play key roles in lipid homeostasis [29]. It was previously shown that PPARG comprises splice variants PPARG1 and PPARG2 [30], and here we showed that different concentrations of TME lead to a significant decrease in PPARG expression, although it remains unclear which splice variants this treatment affects.

CEBPA is a well-known transcription factor that plays vital roles in the differentiation of 3T3-L1 adipocytes. CEBPA activity is intimately associated with that PPARG activity, and CEBPA deficiency in the adipocytes was shown to lead to inhibition of lipid accumulation and differentiation [9], whereas CEBPA alone is unable to promote adipogenesis in the absence of PPARG [31]. Here, we showed that TME treatment resulted in similar, concentration-dependent inhibition of PPARG and CEBPA expression, although PPARG expression was shown to be more affected.

SREBF is a transcription factor that regulates adipocyte differentiation and cholesterol homeostasis by binding to DNA, and it is specifically involved in the regulation of lipogenesis. Previously, it was reported that SREBF1 deficiency inhibits the differentiation of 3T3-L1 adipocytes, and it affects PPARG expression, indicating that this molecule is a key player in PPARG-mediated transcription of adipocyte differentiation-related genes [32]. Our findings demonstrate that TME treatment leads to a decrease in SREBF1 expression, indicating close interactions between these proteins. Furthermore, additional studies are required to identify the specific target of the components of TME among PPARG, CEBP, and SREBF1 and the components of PPARG signaling pathway.

*T. majus* is a rich source of phytochemicals, and it has long been used for long time as an antiseptic, diuretic, purgative, hair tonic, antiscorbutic and anti-inflammatory therapeutic, antihypertensive, and antidepressant. Additionally, it has been used for the treatment of skin diseases, furunculosis, acne, pulmonary disorders, amyotrophic lateral sclerosis, psoriasis, and eczema [33–35]. This plant contains various flavonoids, including kaempferol glucoside and quercetin-3-*O-*glucoside [36]. These flavonoids are known to have diuretic, anti-inflammatory, and anti-acidic effects, and they can inhibit lipid peroxide production and adipocyte differentiation [37–40], which is why we investigated anti-adipogenic properties of *T. majus* flowers. Furthermore, *T. majus* is rich in fatty acids and glucosinolates such as glucotropaeolin, which are heat-stable and possess strong anti-inflammatory and anti-cancer activities [41].

## Conclusions

TME was shown to effectively inhibit the expression of molecules involved in the regulation of lipogenesis and adipogenesis in 3T3-L1 adipocytes, and to reduce triglyceride content and lipid accumulation in these cells. Our future investigations will include the identification of the active component of *T. majus* that exerts the desired anti-adipogenic effects. Our findings, together with the previously reported results, indicate that the edible plant *T. majus* exerts anti-adipogenic effects, and it may be a promising medicinal food item for the prevention and control of obesity.
